# Risk of developing depression among breast cancer patients in Palestine

**DOI:** 10.1186/s12885-022-09420-8

**Published:** 2022-03-21

**Authors:** Dana Sadaqa, Ahlam Farraj, Hani Naseef, Hamza Alsaid, Nimeh Al-Shami, Abdallah Damin AbuKhalil

**Affiliations:** 1 Department of Pharmacy, Faculty of Pharmacy, Nursing and Health Professions, Birzeit University, P.O. Box 14, Birzeit, West Bank Palestine; 2 Department of Internal Medicine, Al-Quds School of Medicine, P.O. Box 17233, East Jerusalem, Palestine

**Keywords:** Breast Cancer, Depression, PHQ 9, Palestine

## Abstract

**Background:**

Breast cancer (BC) is the most common cancer among women. Studies have shown that cancer patients can develop depression impacting their quality of life, treatment outcomes, and survival rates. This study aimed to determine the risk factors, severity and prevalence of depression among females diagnosed with BC in multiple hospitals across Palestine.

**Methods:**

A cross-sectional study was conducted at different cancer treatment centers in Palestine using a previously developed questionnaire consisting of 23 questions to assess the severity of major depressive disorder among females diagnosed with breast cancer. The Patient Health Questionnaire-9 (PHQ-9) was included in the questionnaire.

**Results:**

Out of 223 respondents, 79 (35.4%) have developed moderate to severe depression. Chi-square results revealed that the risk of developing moderate to severe depression was higher among females who suffer from side-effects related to BC treatment (*P* < 0.011), females who knew the BC stage at the diagnosis step (*P* < 0.031), and negative implications on BC patients in Palestine (*P* < 0.009).

**Conclusion:**

Breast cancer patients have an increased risk of developing major depressive disorder. Patient assessment and treatment for depression at the time of BC diagnosis, during the treatment journey, and monitoring after treatment completion is essential for patient quality of life and BC treatment outcomes.

## Background

Breast Cancer (BC) is the most common cancer among women worldwide; according to the World Health Organization (WHO), there were 2.3 million women diagnosed with BC and 685,000 deaths globally in 2020 [1]. In the Eastern Mediterranean Region (EMR), there is an increase in breast cancer incidence ranked as the most prevalent cancer among women [2, 3] combined with the highest mortality rate [2], which is to be attributed to the under developed cancer registration and surveillance systems in the region [3].

In the west bank, BC is also considered the most common type of cancer among women. According to the Palestinian ministry of health (MOH) annual health reports between 2016–2019, 1839 new cases of female BC patients were reported [4–7].

Anatomically, the main components of the female breast are lobules, lactiferous ducts and fatty tissue, in addition to lymph nodes and blood vessels [8]. BC refers to a malignancy originating from these tissues [9]. Furthermore, breast cancer can be classified into non-invasive BC (in situ) or invasive BC according to its relation to the basement membrane [9]. Many reports described these types and their subtypes which have specific properties when it comes to presentation and prognosis [10–12].

Early detection of breast cancer can prevent further disease progression, [13] thus, patient recognition of BC symptoms is crucial. BC symptoms can include a painless lump (which is the most common presenting symptom), lymphadenopathy, nipple changes such as; retraction, discharge and pain.

Diagnosis of breast cancer includes a variety of methods. These include; mammograms, ultrasound and MRI. Utility of these diagnostic modalities can vary according to patient-related factors.

A wide variety of BC treatment options exist depending on patient factors and the disease stage. Early stages (I, II), can be treated with surgery with or without chemotherapy. Surgical management of BC can range from lumpectomy to radical mastectomy [14]. Radiation therapy is often used after breast surgery to prevent recurrence [15], while chemotherapy can be utilized after surgery (Adjuvant chemotherapy) or -in some cases- before surgery (neoadjuvant), especially when the tumor size is larger than 5 cm (stage III) [16].

There is evidence that BC women are at high risk of developing depression within the first year of diagnosis [17, 18]. A review study showed that 41% of newly- diagnosed BC patients suffer from distress while 11% suffer from major depressive disorder [19] and another study found that 10 to 32% of young patients diagnosed with BC develop depression more often [20].

Depression is one of the most common psychiatric illnesses, caused by Serotonin and Nor-epinephrine deficiency [10], but in cancer patients other pathogenic factors have been identified where major depressive disorder (MDD) has been mostly linked to an increase in cytokines including Interleukin (IL)-6, which further depletes the amino acid tryptophan and induces indolamine 2,3 dioxygenase (IDO) enzyme [21]. And due to an increase in these inflammatory mediators as well as oxidative stress, glutamatergic excitotoxicity develops [22]. Patients suffering from depression can present with different symptoms such as: feelings of guilt and worthlessness, sleep and appetite disturbances, loss of energy and anhedonia. Depression can range from mild to severe depending on the symptoms and the impact on patients’ quality of life (QoL) [23].

According to the fifth Diagnostic and Statistical Manual (DSM-5) of mental disorders for depression, Symptoms should be present for 2 weeks or more to make a diagnosis of depression, not attributable to psychological effects of a substance or another medical condition [10]. PHQ-9 can also be used to screen for depression caused by a medical condition, such as CHF and cancer.

Many studies were conducted to determine factors associated with developing depression among BC patients. Receiving chemotherapy treatment [18], breast mastectomy surgery[19], social support [23] not attending any psychosocial support programs [24] and lower educational level [25] are all factors associated with high risk for developing depression. At the same time, it is controversial whether the stage of disease at diagnosis is directly related to development of depression or not [19]. Knowledge of breast cancer diagnosis and information provision has been associated with lower risk of depression due to the improvement of patient satisfaction and quality of life, based on previous studies [26, 27], which is counteractive to the results obtained in this study.

In Palestine, the prevalence and severity of depression among BC patients during diagnosis or treatment have not been addressed or reported in the annual health report of the ministry of health. However, many studies suggested a direct relationship between depression and cancer and its negative impact on patients’ quality of life and well-being. Therefore, our research aimed to evaluate the prevalence, the risk factors and severity of depression among females diagnosed with breast cancer in Palestine. Furthermore, our study is conducted to evaluate the relationship between social support and its role in preventing, decreasing severity as well as frequency of depressive episodes.

## Methods

### Study participants and sample size

The sample size was calculated based on the number of patients diagnosed with breast cancer in the West Bank between the years 2016–2019, which was 1839 cases [4–7]. The calculated study sample was 223 patients with a 95% confidence interval and a 5% margin error [28].

Patients were recruited from cancer treatment centers at Dunya Diagnostic Center for BC, oncology departments in Augusta Victoria Hospital, Istishari Arab Hospital, Beit Jala Hospital, and Palestinian Medical Complex hospital. Females aged 30–70 years who were diagnosed with primary BC within the last year in the West Bank and Jerusalem were included in the study. Secondary BC cases, where cancer has originated from another site and metastasized to the breast were excluded in the study. Participants were classified based on their BC treatment plan chemotherapy, surgery (lumpectomy, mastectomy, lymph node dissection) and radiotherapy and eligible patients were chosen based on hospital clinical records.

#### Study design and data collection

Data collection took place between January and April 2021. 318 questionnaires were distributed to the previously mentioned locations, but due to limited time, only 223 samples were filled out. Researchers or healthcare professionals have interviewed the patients and filled the questionnaires after obtaining verbal consent*.* Verbal informed consent was obtained by each participant before enrollment and this verbal consent procedure was approved by Ethics Committee of the Research Ethics Committee at the Faculty of Pharmacy, Nursing and Health Professions, Birzeit University [Approval number BZU-PNH-2002].

A questionnaire was constructed after reviewing the literature [29, 30], written in English, reviewed by nurses, oncologists, and clinical pharmacists; their comments and expertise were taken into consideration for modification of the questionnaire before data collection, also feedback was received from psychiatrists and psychologists. The questionnaire was then translated into Arabic by an expert then distributed for patients. The questionnaire consisted of two parts; the first part is made up of fourteen questions, which included socio-demographic information including age of the patient, residency region and disease-related questions including age at diagnosis, knowledge of disease stage and diagnosis, treatment plan, treatment side-effects, availability and source of psychological support, patient’s view on psychological support and negative consequences of the disease. The answers were displayed as multiple-choice categories. The second part of the questionnaire included the PHQ-9 diagnostic assessment questionnaire for MDD. PHQ-9 is considered a screening tool for depression that consists of 9 questions to assess symptoms of depression in the last two weeks, including sleep disturbance, appetite changes, fatigue, loss of interest, suicidal thoughts, feelings of guilt, difficulty concentrating, and excessive movement or speaking. Each question is assigned a score from zero to three. A value of zero was assigned to no symptoms and a value of three for daily symptoms.

### Statistical analysis

Data was inserted into google forms, cleared, transferred, and analyzed using Statistical Package for Social Sciences (SPSS) version 22. Four treatment groups including chemotherapy as part of their treatment plan, were resorted and combined to compare the risk of developing depression in patients receiving chemotherapy vs. patients not receiving chemotherapy. The PHQ9 score was used to assess depression and the reliability coefficient Cronbach’s alpha factor was measured and revealed a good internal consistency (Cronbach α = 0.753).

Patients’ score was calculated then classified as healthy- mildly depressed if the score was less than 10 and as moderate- severely depressed if the score was 10 or more.

Descriptive statistics (mean, standard deviation, and frequencies) were calculated for both BC and depression-related questions. Pearson Chi-square test was performed at a 95% confidence level to identify the potential associated factors between BC disease and the PHQ-9 dichotomous scale.

## Results

A total of 223 participants answered the questionnaire, 193 (86.5%) were West Bank inhabitants and 73 (32.7%) were less than 40 years when they were diagnosed.

According to the PHQ-9 score in Table 1, 79 (35.4%) women suffered from moderate-severe depression symptoms. In addition, sleep disturbance and low energy were the most common symptoms reported (mean=1.46), while thoughts about death, or hurting themselves were the lowest reported symptoms, followed by feeling bad about themselves (mean=0.26, 0.39), respectively.

**Table 1:** PHQ-9 scale and score for depression (N 223)

**Symptoms**	Never n (%)	Several days n (%)	half of the days or more n (%)	almost every day n (%)	Mean	Standard deviation
Little interest or pleasure in doing thing	47 (21.1)	105 (47.1)	51 (22.9)	20 (9.0)	1.20	0.873
Feeling down, depressed, or hopeless	79 (35.4)	102 (45.7)	24 (10.8)	18 (8.1)	0.91	0.884
Trouble falling or staying asleep, or sleeping too much	33 (14.8)	102 (45.7)	41 (18.4)	47 (21.1)	1.46	0.985
Feeling tired or having little energy	24 (10.8)	112 (50.2)	47 (21.1)	40 (17.9)	1.46	0.909
Poor appetite or overeating	42 (18.8)	107 (48.0)	45 (20.2)	29 (13.0)	1.27	0.916
Feeling bad about yourself	159 (71.3)	49 (22.0)	6 (2.7)	9 (4.0)	0.39	0.733
Trouble concentrating on things, such as reading the newspaper	82 (36.8)	97 (43.5)	22 (9.9)	22 (9.9)	0.93	0.927
Moving or speaking so slowly that other people could have noticed	114 (51.1)	77 (34.5)	19 (8.5)	13 (5.8)	0.69	0.859
Thoughts that you would be better off dead, or of hurting yourself in some way	183 (82.1)	28 (12.6)	7 (3.1)	5 (2.2)	0.26	0.624

Table 2 showed that 30 (39%) of patients aged 40-49 years had the highest risk of developing moderate-severe depression, and 69 (35.8%) developed moderate-severe depression in the group of West Bank inhabitants.

**Table 2:** Patients’ age residency region vs Depression (N 223)

Variable	Participants n (%)	Asymptomatic-mild depression	Moderate-severe depression	*P*- value
Residency region				
West Bank	193 (86.5%)	124 (64.2%)	69 (35.8%)	0.797
Gaza strip	30 (13.5%)	20 (66.7%)	10 (33.3%)	
Age at diagnosis				
< 40	73 (32.7%)	48 (65.8%)	25 (34.2%)	0.714
40- 49	77 (34.6%)	47 (61.0%)	30 (39.0%)	
50 or more	73 (32.7%)	49 (67.1%)	24 (32.9%)	

Table 3 showed that only 35 (15.7%) of respondents knew their stage at diagnosis. For most patients, chemotherapy and surgery were part of their treatment plan. 196 (87.9%) of patients had surgery, and 203 (91.0%) of patients were on chemotherapy. As shown in Fig. 3, among women who underwent chemotherapy treatment, 88 (39.5%) suffered from many side effects, and 164 (73.5%) had alopecia.

**Table 3:** Knowledge of Diagnosis, Stage, Treatment plan and surgery type vs Depression (N 223)

Variable	Participants n (%)	Asymptomatic-mild depression	Moderate-severe depression	*P*- value
**Knowledge of BC stage or type**				
** Yes**	35 (15.7%)	17 (48.6%)	18 (51.4%)	0.031
** No**	188 (84.3%)	127 (67.6%)	61 (32.4%)	
**Treatment plan**				
** + Chemo**	203 (91.0%)	130 (64.0%)	73 (36.0%)	0.595
** -Chemo**	20 (9.0%)	14 (70.0%)	6 (30.0%)	
**Surgery Type**				
** Partial (± LN)**	119 (53.4%)	74 (62.2%)	45 (37.8%)	0.147
** Total (± LN)**	77 (34.5%)	48 (62.3%)	29 (37.7%)	
** No surgery**	27 (12.1%)	22 (81.5%)	5 (18.5%)	
**Number of side effects due to chemotherapy**				
** 0 to 3**	135 (60.5%)	96 (71.1%)	39 (28.9%)	0.011
** 4 to 7**	88 (39.5%)	48 (54.5%)	40 (45.5%)	
**Chemotherapy-induced alopecia**				
** Hair loss**	164 (73.5%)	101 (61.6%)	63 (38.4%)	0.120
** No Hair loss**	59 (26.5%)	43 (72.9%)	16 (27.1%)	

** + Chemo: treatment plan include chemotherapy, -Chemo: treatment plan does not include chemotherapy, Partial (± LN): partial mastectomy with or without lymph node removal, Total (± LN) total mastectomy with or without lymph node removal**

The Chi- square test revealed a significant association between knowing the BC stage and the possibility of developing depression (*p*- value = 0.031). Women knowing their disease type or stage (51.4%) were more likely to develop moderate- severe depression compared to patients who didn’t (32.4%).

Respondents who reported having many side effects after chemotherapy treatment (45.5%) were significantly more likely to develop moderate- severe depression (*p*- value = 0.011) compared to those who reported fewer side effects (28.9%). No significant associations were found between factors total mastectomy surgery or hair loss and the possibility of developing depression.

**Fig. 1:**
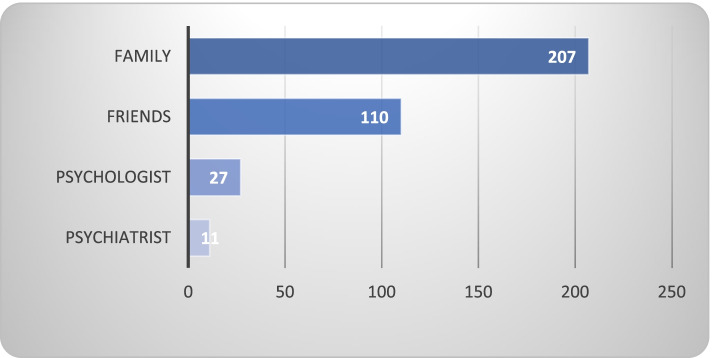
Psychological support sources for BC patients (N 223)

As shown in Fig. 1, the majority of respondents reported receiving social support to cope with their disease, 207 reported that their family members were the primary source of support, while 110 of patients reported receiving support by friends, 27 patients received support by a psychologist and only 11 patients received support by a psychiatrist. 3 (1.3%) of them were diagnosed with depression and treated with an antidepressant, and (8%) received counseling by a psychologist. Nevertheless, the PHQ-9 scale revealed that (35.4%) were at risk of suffering from moderate-severe depression and needed psychiatrist support and treatment.

**Fig. 2:**
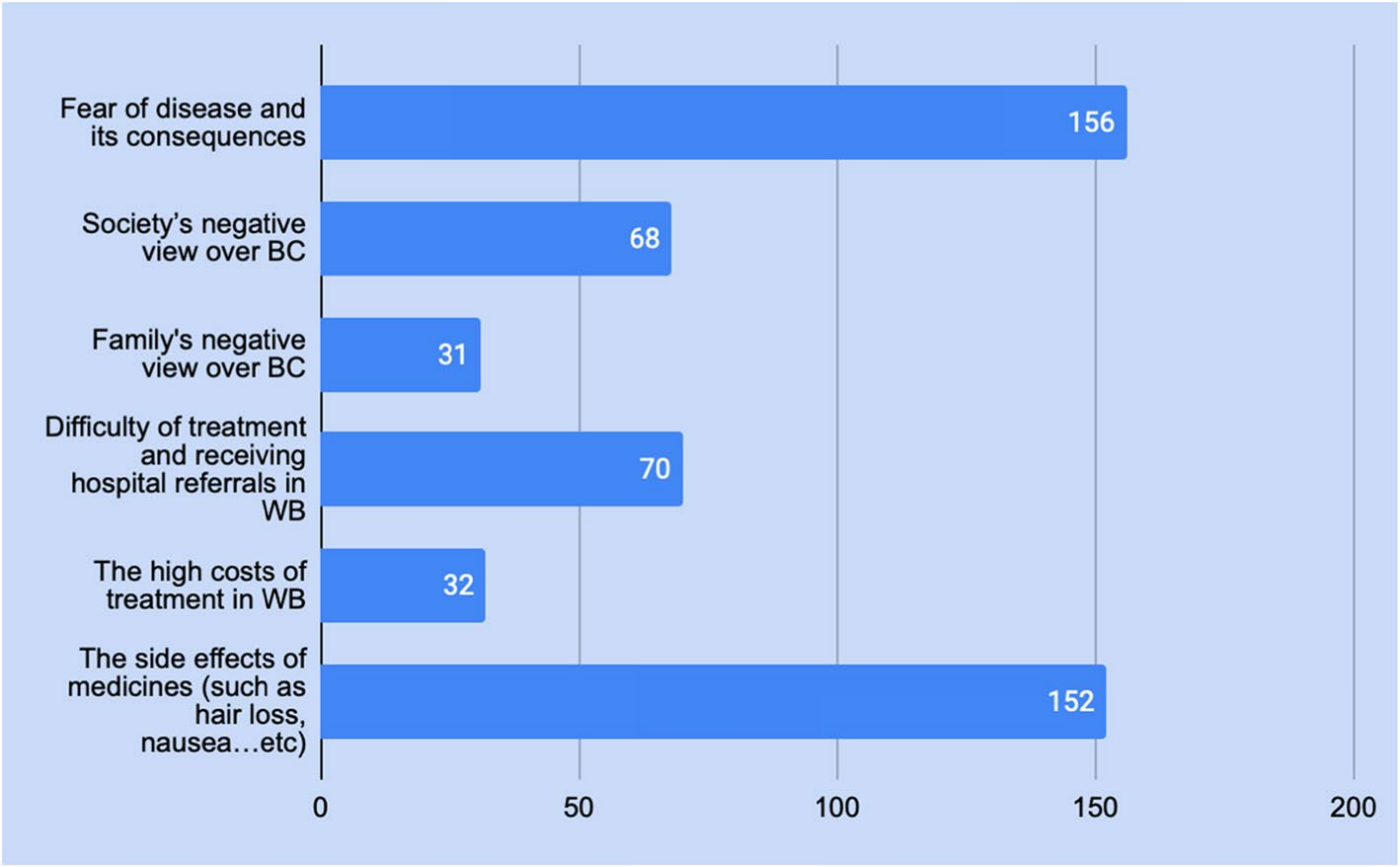
Restraints on BC patients in Palestine

Cancer, in general, has many restraints on a patient’s life. 156 participants revealed that they are afraid of the disease itself and its consequences and 152 revealed that the chemotherapy treatment side effects are one of the main negative consequences on their life. The other factors are shown in Fig. 2

**Fig. 3:**
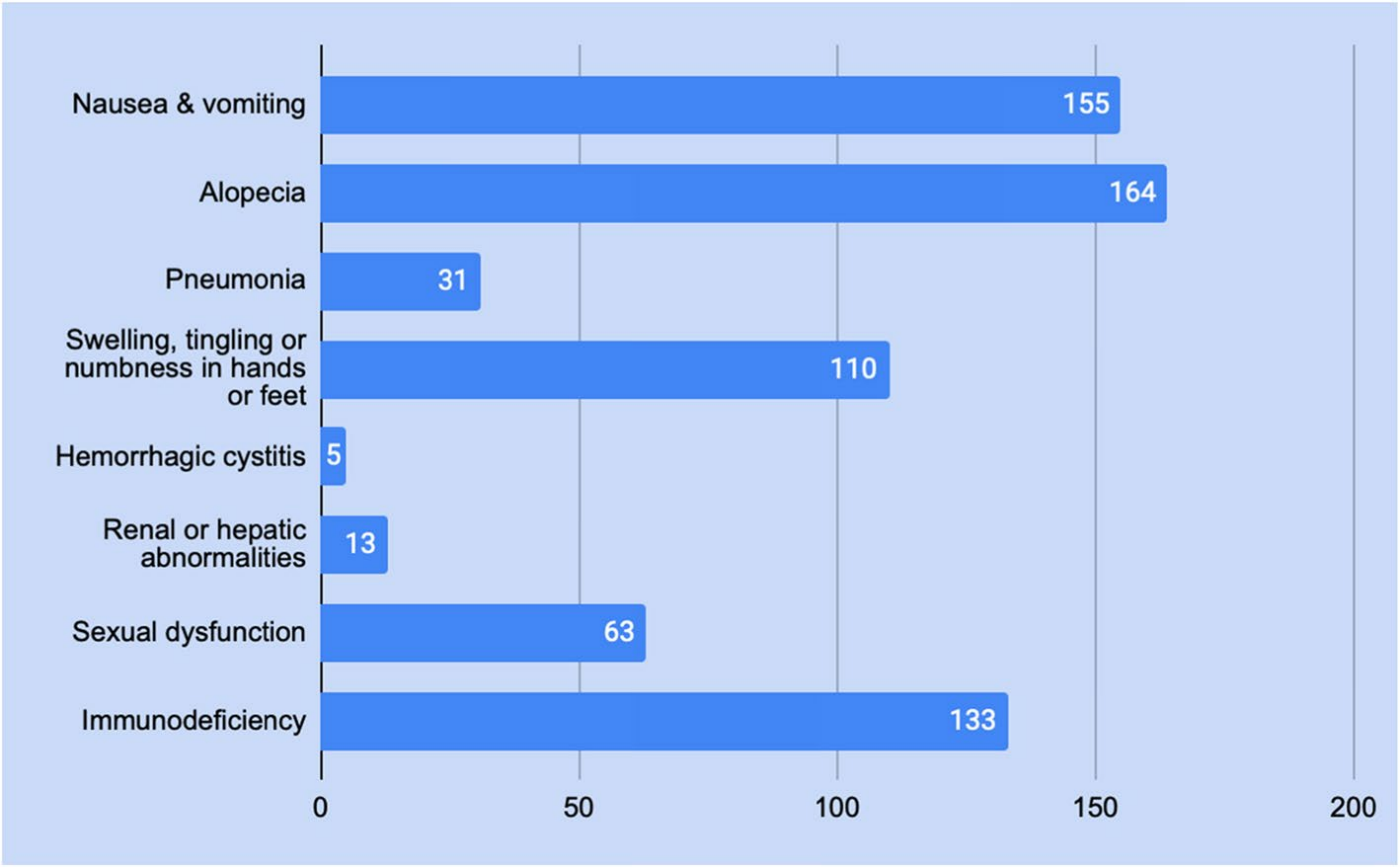
Side effects of breast cancer treatment

Figure 3, BC: patients undergoing chemotherapy treatment complained of many side effects, the most common (164 patients) of which was facial and scalp hair loss, followed by nausea and vomiting (155 patients).

Table 4 shows the relationship between difficulties and side effects due to treatment, medical referrals, and unavailability of support with the possibility of developing depression. However, the results revealed a relation only between facing adverse impacts and the possibility of developing moderate-severe depression (*P*-value = 0.009). Patients with many restraints (66.7%) are more likely to develop moderate-severe depression than those with fewer restraints (33.2%).

**Table 4:** Psychological support and negative implications VS Depression in breast cancer patients (N 223)

Variable	Participants n (%)	asymptomatic-mild depression	Moderate-severe depression	*P*- value
Receiving support (sources)				
Little ≤ 2	102 (45.7)	63 (61.8)	39 (38.2)	0.421
Many > 2	121 (54.3)	81 (66.9)	40 (33.1)	
Supported by psychologist counseling				
Yes	27 (12.1)	19 (70.4)	8 (29.6)	0.502
No	196 (87.9)	125 (63.8)	71 (36.2)	
Number of limitations due to disease				
Little ≤ 3	208 (93.3)	139 (66.8)	69 (33.2)	0.009
Many > 3	15 (6.7)	5 (33.3)	10 (66.7)	
Having treatment difficulty and receiving hospital referrals				
Yes	70 (31.4)	44 (62.9)	26 (37.1)	0.717
No	153 (68.6)	100 (65.4)	53 (34.6)	
Fear of the disease and its consequences				
Yes	156 (70.0)	97 (62.2)	59 (37.8)	0.254
No	67 (30.0)	47 (70.1)	20 (29.9)	
Side effects of medicines affects patients' life				
Yes	144 (64.6)	94 (61.8)	58 (38.2)	0.212
No	79 (35.4)	50 (70.4)	21 (29.6)	

The relationship between fearing the disease and having medication side effects that negatively affect patients’ lives, treatment difficulties or unavailability of support, and depression appears to have increased the risk of developing depression but results were not statistically significant.

As shown in Fig. 2, 24% of all participants didn’t receive psychological support. Of those, 91.7% (22% overall) thought they needed it, while only 8.3 (2% overall) thought it was not essential.

## Discussion

Depression was a common and significant co-morbidity among BC patients who participated in the study. The prevalence of depression in Palestine among breast cancer patients was 35.4% which is much higher compared to nearby countries, as in Jordan (30.2%) [31] and Lebanon (24.7%) [32], and lower than Egypt (68.6%) [33].

There was no significant difference in depression rates between the West Bank and Gaza dwellers although it was expected to have more cases of moderate-severe depression among Gaza dwellers, since this population group might suffer more difficulties in terms of hospital referrals and access to health care. Reports indicating and explaining differences between geographical areas are lacking, thus the exact causes of possible differences is yet not clear. we believe that this can be multifactorial including differences in socioeconomic status and educational level, we believe that more future studies need to be done to inspect these differences.

The prevalence of depression was not significantly different between different age groups. This finding is inconsistent with Adam and Koranteng’s study, where younger patients may be at higher risk of developing depression due to BC's psychological impact on their physical appearance and quality of life [34]. These findings may be related to a higher percentage of elderly patients who participated in study, leading to a decrease in the power to assess risk of developing depression in younger age group, as opposed to previous literature. [19] In addition, neither mastectomy nor alopecia was associated with increasing the risk of depression development compared to what has been approved by other studies [20, 29, 33, 34], but patients who had partial or total mastectomy had higher risk of developing moderate to severe depression compared to patients who didn’t undergo mastectomy surgery. The major surgical treatment of BC usually includes partial or total mastectomy. If lymph nodes are invaded, they are removed as well [28]. Also, the social and cultural belief system and women’s physical appearance might differ between different populations and cultures. We noted that the psychological impact of chemotherapy-induced alopecia was high but statistically insignificant, this could be explained by the fact that many of Palestinian females cover their hair.

Knowledge of BC stage at diagnosis and the exact BC type increased the risk of developing moderate-severe depression. This finding could be due to the patients’ ability to read more about their disease online and know the risk of recurrence and prognosis of their disease compared to other people who only knew very little detail about their illness [20]. It was also noticed that after interviewing patients at Augusta Victoria Hospital, many of them did not receive high school education. Their religious and spiritual beliefs played an essential role in coping with their illness.

Side effects related to chemotherapy, such as alopecia, fatigue, pain, nausea, vomiting, and tiredness, are risk factors for developing depression and negatively affect BC patients’ quality of life and prognosis. This finding was supported by other studies where chemotherapy has increased the risk of depression and anxiety [31, 35]. In addition, chemotherapy’s adverse effects remain almost one-month post-treatment, whereas adverse effects of radiotherapy usually decline throughout therapy [32]. Appropriate patient assessment and selecting chemotherapy agents based on patient comorbidities and organ functions are factors that should be addressed at every patient encounter. Furthermore, optimizing medication therapy to treat or prevent side effects will improve patient chemotherapy treatment experience and decrease risk factors for developing depression-related to side effects such as appropriate measures to prevent and treat nausea, vomiting, and pain.

Diagnosis of cancer carries many negative implications on BC patients in Palestine such as fear due to false beliefs, and lack of education despite the development of medicine and treatment options in the West Bank hospitals nowadays compared to many years ago when mortality rate was higher [36]. As a result, the majority of patients suffered from 4fear of the disease and its consequences. In addition, one-third of the respondents complained of the difficulty of long-distance travel and the lengthy approval for hospital referral to a cancer treatment center. Currently, there are insufficient hospitals in West Bank and Gaza for cancer treatment, which might delay treatment.

**Fig. 4:**
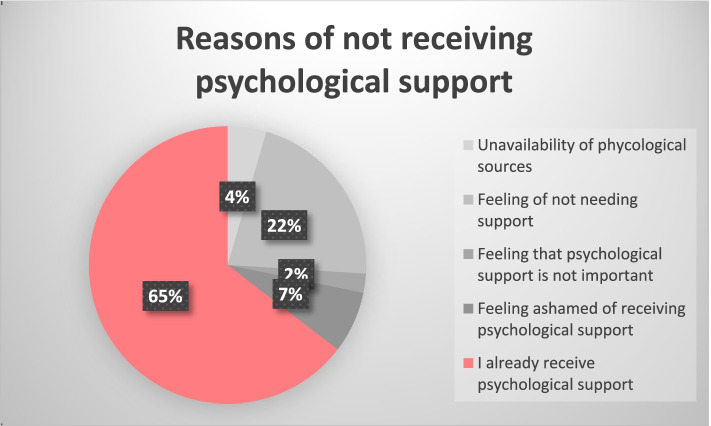
Reasons for not receiving psychological support (N 223)

As shown in Fig. 4, the most common reason among patients for not receiving psychological support from a healthcare professional, was that they felt it was unnecessary and not needed and family support is sufficient. Furthermore, seeking psychological support and mental illness help can be difficult among this population due to mental illness stigmatization. In addition, continuous psychological support and assessment at the time of diagnosis, during and following treatment are not considered an integral part of treatment protocols in all centers. As a result, mental illnesses can be miss diagnosed and undertreated in the West Bank [37]. Patients who received psychologist support had a lower risk of developing moderate to severe depression than patients who did not. Receiving adequate psychological support is essential to reduce the risk of depression in breast cancer patients, as shown in Table 4 patients who received support from more than 2 sources had 5% reduced risk of developing moderate-severe depression symptoms compared to patients who received support from 2 or less sources, it is also mentioned in a previous study relating adequate family support to reduced risk of developing depression [38], as well as another study where a 1-year of participation in cancer support, improved well-being of the patient was observed [39].

Suicide ideation and self-harm are unusual behaviors in Palestine mainly because of religious beliefs. That fact is reflected on the PHQ-9 scale question about suicidal thoughts which had the lowest mean.

Our main study limitation is that we had difficulty assessing the stage of breast cancer which might be related to an increased risk of developing depression in patients, and that can be studied in future research. In addition, the educational level of the patients should also be assessed in future studies related to this topic. Also, a bigger sample size can be beneficial in assessing the relationship between the studied factors and depression severity.

## Conclusion

Depression is a common comorbidity among breast cancer patients in Palestine. The psychological impact of the disease, the side effects of chemotherapy, the lack of access to healthcare, and cancer treatment centers are all risk factors that increase the severity of this comorbidity. In our study, 35.4% female breast cancer patients have been initially screened for depression through the PHQ-9 questionnaire and would need further evaluation by a psychiatrist for final diagnosis of depression, compared to only 1.3% who were previously diagnosed with depression by psychiatrists. Furthermore, cancer treatment centers need to incorporate mental illness assessment, treatment, and support as part of their treatment plans and protocol to treat and prevent depression among breast cancer patients.

## Data Availability

The datasets generated during and analyzed during the current study are not publicly available due to privacy and confidentiality of the patients, and the stigma concerning psychological illness in the community, but are available from the corresponding author on reasonable request.

## Abbreviations

PHQ-9: Patient-Health questionnaire 9
BC: Breast cancer
MDD: Major Depressive Disorder
QoL: Quality of life
MOH: Ministry of Health
DCIS: Ductal carcinoma in situ
LCIS: Lobular carcinoma in situ
IDC: Invasive ductal carcinoma
ILC: Invasive lobular carcinoma
DSM IV: Diagnostic and statistical manual
SPSS: Statistical Package for Social Sciences
